# Hyderabad Chloroform Commission

**Published:** 1890-05

**Authors:** 


### SELECTIONS.

### Hyderabad Chloroform Commission.

One of the most important and satisfactory series of investigations in the province of medicine, is that which has recently been completed by the Hyderabad Chloroform Commission. The commission owed its existence to Surgeon-Major Lawrie's veneration for his late teacher, Professor Syme, and his desire to prove the correctness of Syme's teaching that chloroform might be administered with perfect safety, provided that the administrator watched the respiration with sufficient care. Accordingly, some time ago, at Dr. Lawrie's suggestion, the Nizam's government appointed a commission which conducted a series of experiments and arrived at the conclusion that chloroform always arrests the respiration before the heart. But the facts brought forward seemed insufficient to overthrow the conclusions of many observers, both in Europe and elsewhere, whose researches seemed to prove that one of the dangers from chloroform was paralysis of the heart.

Dr. Lawrie, therefore, proposed a second commission, to which the London *Lancet* should send a representative, and for this purpose the Nizam's Government generously offered the sum of £1,000. As our readers already know, the *Lancet* selected Dr. Lauder Brunton and, on his arrival at Hyderabad on Oct. 22, 1889, a second commission was immediately formed, consisting of Surgeon-Major Lawrie, as President, and Drs. Lauder Brunton, Bomford and Rustomji, as members. Their complete report appeared in the *Lancet* for January 18, 1890.

The experiments were of two kinds, those of one group being made without recording apparatus, and being intended to ascertain what influence is exerted by various conditions upon the relation between the stoppage of heart and of respiration, and the limits within which artificial respiration and other means of resuscitation are useful. The second group consisted of experiments with recording apparatus, and were made for the purpose of ascertaining the effect of various conditions upon the heart and blood pressure. In the first group chloroform was given in all sorts of ways, alone or with morphine, atropine, and strychnine, to animals healthy and diseased, fasting or replete. The result was invariable: in every case the respiration stopped before the heart, sometimes a long time before it. But the effect of partial asphyxia in causing the heart to stop very soon after the respiration is deserving of particular attention.

The second group of experiments on heart and blood pressure was made with apparatus arranged in such a way that the whole experiment could be recorded from beginning to end in such a compass as to admit of photographic reproductions in its entirety. This was managed by recording the general blood pressure on a slowly revolving drum, and taking at intervals a tracing on a second drum, revolving with sufficient rapidity to show each beat of the pulse. About one hundred and fifty experiments were made in this way, and the influence of everything that seemed likely to affect the blood pressure during chloroform narcosis was ascertained. Particular attention was directed to the production of shock or syncope, and to the effect of chloroform itself on the heart and blood-pressure in healthy animals, and also in cases where fatty degeneration of the heart and other organs had been produced by the previous administration of phosphorus. The results of these experiments were unexpected. It was found to be exceedingly difficult to affect the heart reflexly, and recourse was therefore had to direct stimulation of the vagus, by which the heart can be slowed or stopped completely. Instead of causing the death of the animal, however, it appeared rather to be a safeguard, preventing the anæsthetic from being conveyed in too great quantities to the nerve centres.

In the *Lancet's* note to one of Dr. Brunton's telegrams, and referred to in the *Reporter*, Jan. 4, 1890, attention was called to the change which his views had undergone. From the present report it may, however, be seen that the discrepancy between the views of different schools arises from the fact that sufficient consideration has not been given to the conditions under which the chloroform is given. Although it may paralyze the heart if applied directly to it, yet this condition does not occur in practice, for here it is neither applied to that organ nor yet is it blown forcibly into the lungs. It is inhaled by the patient, and when this is the case it stops the respiration before the heart. The practical outcome of the research would appear to be that deaths from chloroform are not inevitable. They are, therefore, preventable, and by due care in its administration they may be with certainty avoided. The conclusions of the commission are sweeping, and without abundant evidence can not be accepted. Fortunately the generosity of the Nizam's Government has not been limited to the appointment and payment of the commission, but by well-timed liberality it has secured the permanent utility of the work done by it, for it has had every tracing photographically reproduced, and will present a copy of the complete work to all the principal medical libraries throughout the world.

The following are the practical conclusions which the commission think may fairly be deduced from the experiments recorded in their report:

I. The recumbent position on the back and absolute freedom of respiration are essential.

II. If during an operation the recumbent position on the back can not, from any cause, be maintained during chloroform administration, the utmost attention to the respiration is necessary to prevent asphyxia or an over dose. If there is any doubt whatever about the state of respiration, the patient should be at once restored to the recumbent position on the back.

III. To insure absolute freedom of respiration, tight clothing of every kind, either on the neck, chest or abdomen, is to be strictly avoided; and no assistants or bystanders should be allowed to exert pressure on any part of the patient's thorax or abdomen, even though the patient be struggling violently. If the struggling does occur, it is always possible to hold the patient down by pressure on the shoulders, pelvis or legs without doing anything which can by any possibility interfere with the free movements of respiration.

IV. An apparatus is not essential, and ought not to be used, as, being made to fit the face, it must tend to produce a certain amount of asphyxia. Moreover, it is apt to take up part of the attention which is required elsewhere. In short, no matter how it is made, it introduces an element of danger into the administration. A convenient form of inhaler is an open cone or cap with a little absorbent cotton inside at the apex.

V. At the commencement of inhalation care should be taken, by not holding the cap too closely over the mouth and nose, to avoid exciting, struggling or holding the breath. If struggling or holding the breath do occur, great care is necessary to avoid an over-dose during the deep inspirations which follow. When quiet breathing is ensured as the patient begins to go over, there is no reason why the inhaler should not be applied close to the face; and all that is then necessary is to watch the cornea and to see that the respiration is not interfered with.

VI. In children, crying ensures free admission of chloroform into the lungs; but as struggling and holding the breath can hardly be avoided, and one or two whiffs of chloroform may be sufficient to produce complete insensibility, children should always be allowed to inhale a little fresh air during the first deep inspirations which follow. In any struggling persons, but especially in children, it is essential to remove the inhaler after the first or second deep inspiration, as enough chloroform may have been inhaled to produce deep anæsthesia, and this may only appear, or may deepen, after the chloroform is stopped. Struggling is best avoided in adults by making them blow out hard after each inspiration during the inhalation.

VII. The patient is, as a rule, anæsthetized and ready for the operation to be commenced when unconscious winking is no longer produced by touching the surface of the eye with the tip of the finger. The anæsthetic should never, under any circumstances, be pushed till the respiration stops; but when once the cornea is insensitive the patient should be kept gently under, by occasional inhalations, and not be allowed to come out and renew the stage of struggling and resistance.

VIII. As a rule, no operation should be commenced until the patient is fully under the influence of the anæsthetic, so as to avoid all chance of death from surgical shock or fright.

IX. The administrator should be guided as to the effect entirely by the respiration. His only object, while producing anæsthesia, is to see that the respiration is not interfered with.

X. If possible, the patient's chest and abdomen should be exposed during chloroform inhalation, so that the respiratory movements can be seen by the administrator. If anything interferes with the respiration in any way, however slight, even if this occurs at the very commencement of the administration, if breath is held, or if there is stertor, the inhalation should be stopped until the breathing is natural again. This may sometimes create delay and inconvenience with inexperienced administrators, but experience will make any administrator so familiar with the respiratory functions under chloroform that he will, in a short time, know almost by intuition whether any thing is going wrong, and be able to put it right without delay before any danger arises.

XI. If the breathing becomes embarrassed the lower jaw should be pulled or pushed from behind the angles, forward, so that the lower teeth protrude in front of the upper. This raises the epiglottis and frees the larynx. At the same time it is well to assist the respiration artificially until the embarrassment passes off.

XII. If, by any accident the respiration stops, artificial respiration should be commenced at once, while an assistant lowers the head and draws forward the tongue with catch-forceps, by Howard's method, assisted by compression and relaxation of the thoracic walls. Artificial respiration should be continued until there is no doubt whatever that natural respiration is completely re-established.

XIII. A small dose of morphia may be injected subcutaneously before chloroform inhalation, as it helps to keep the patient in a state of anæthesia in prolonged operations. There is nothing to show that atropine does any good in connection with the administration of chloroform, and it may do a great deal of harm.

XIV. Alcohol may be given with advantage before operations under chloroform, provided it does not cause excitement, and merely has the effect of giving a patient confidence and steadying the circulation.

The commission has no doubt whatever that, if the above rules be followed, chloroform may be given in any case requiring an operation with perfect ease and absolute safety so as to do good without the risk of evil.

